# A classroom-based intervention targeting working memory, attention and language skills in 4–5 year olds (RECALL): study protocol for a cluster randomised feasibility trial

**DOI:** 10.1186/s40814-019-0468-8

**Published:** 2019-06-24

**Authors:** Anita Rowe, Jill Titterington, Laurence Taggart

**Affiliations:** Institute of Nursing and Health Research, Ulster University, Shore Road, Newtownabbey, Antrim BT37 0QB Northern Ireland

**Keywords:** Working memory, Attention, Language, Intervention, School, Children, Feasibility, Study protocol

## Abstract

**Background:**

There is international recognition of the need for creative, classroom-based interventions to support children at risk of low academic achievement and well-being, due to poor attention and language skills on school entry. Working memory (WM) is a cognitive skill that is strongly associated with attention and language skills. There has been speculation that WM training, embedded within typical educational activities, may improve children’s WM skills and produce transfer effects to real-world skills such as attention and language. However, little is known about the effectiveness of this approach.

‘Recall to Enhance Children’s Attention, Language and Learning’ (RECALL) is a novel, 6-week, classroom-based intervention targeting WM, attention and language skills in 4–5 year olds. RECALL was co-produced with health professionals, teachers and parents. This protocol describes the rationale, methods and analysis plan for a proposed cluster randomised feasibility trial of this RECALL programme.

**Methods:**

This is a three-arm, cluster randomised feasibility trial comparing RECALL to an existing programme (active control), and no-intervention (education as usual). We will recruit six schools in socially disadvantaged areas in one region of the UK. Two schools will be randomly allocated to each arm of the trial. In each school, one class of children (ages 4-5 years) of approx. 30 children will be involved in this study. Ten children in each class will be sampled purposefully for outcome measurement including: standardised assessments of WM, language and attention skills; teacher ratings of attention; and parent ratings of functional communication skills. These will be administered at baseline and 1-week post-intervention in order to test the acceptability of the measures. A process evaluation using semi-structured interviews with participants will explore the acceptability of RECALL and the procedures employed in this trial.

**Discussion:**

This feasibility study will explore the acceptability of RECALL to the health professionals and teachers who will deliver it and inform the optimal design of the programme. The inclusion of an active control group and the blinding of outcomes assessors enhance rigour in this study. The findings will determine whether this study can be scaled-up into a definitive cluster randomised trial to evaluate the effectiveness of RECALL.

**Trial registration:**

ISRCTN13633886. Registered 7 Sept 2018.

## Background

Internationally, the need for classroom interventions that break cycles of academic underachievement, unemployment and poor mental and physical health is widely acknowledged [1]. In areas of low socio-economic status (LSES), high proportions of children present with impoverished language skills on school entry, and are subsequently at risk of poor school performance [2–4]. To address this, Speech and Language Therapy and other health services provide early intervention in schools through collaborative, classroom-based approaches. However, there is a lack of research-based evidence for the effectiveness of such interventions [5–7]. There is now a need for creative, therapeutic interventions to support this population [8, 9].

For children from LSES backgrounds, low language is often associated with cognitive difficulties [10, 11]. Working memory (WM) is a cognitive skill reflecting the ability to hold in mind and mentally manipulate information over short periods of time in the face of distraction [12–14]; it is strongly associated with attention skills [15–17] and language acquisition [18]. The implication of the symbiotic relationship between WM, attention and language [19] is that targeting WM as an underlying skill may produce improvements in these real-world skills [20].

Most research into the effectiveness of WM interventions has investigated computerised training packages (e.g. Cogmed 2005 [21]). The therapeutic value of this approach has been debated due to the consistently inconsistent evidence for transfer effects, i.e. the generalisation of positive effects on trained tasks to other untrained tasks [22]. In the WM literature, transfer effects have been differentiated into near-transfer and far-transfer. Near-transfer refers to improvements in untrained tasks that are similar to those trained, e.g. improvements in a visuospatial WM task following training on a verbal WM task [23, 24]. Far-transfer refers to enhanced performance in tasks quite different from those trained but which are deemed to be dependent on WM, e.g. improvement on real-world skills including language, literacy, numeracy and the ability to pay attention in class following WM training [23, 24]. The presence of near-transfer effects associated with an intervention is an essential criterion to corroborate any far-transfer effects identified [25].

On the basis that treatment effects are likely to transfer to activities with overlapping features to the trained task [26], there have been calls for WM training to be embedded within the typical activities in which benefits are needed [27, 28]. However, there has been limited research into the effectiveness of WM interventions applied with young children in everyday contexts. Furthermore, to date, the literature has focused on the cognitive benefits of WM training, and the individual differences that may moderate or mediate training and transfer effects [29, 30]. There has been little consideration of the contextual factors associated with the delivery of WM interventions in real-life settings such as schools. It has been suggested that controlling the quality, dose and fidelity of WM training in the classroom setting is challenging [29], but there has been a lack of empirical research into the barriers and facilitators of implementing WM interventions in schools.

To address this, we have developed the ‘Recall to Enhance Children’s Attention Language and Learning’ (RECALL) programme. RECALL is a theoretically underpinned, evidence-based intervention that targets WM, attention and language skills in 4–5-year-old children through group and whole-class activities over a 6-week period. It is designed to be delivered by teachers and teams of health professionals that are commissioned to reduce barriers to learning in one region of the United Kingdom (UK), Northern Ireland [31]. The Regional Integrated Support for Education (RISE) teams include speech and language therapists (SLTs), occupational therapists (OTs), physiotherapists (PTs) and social, emotional and behavioural specialists (SEBs). They support children aged between 3 and 8 years (nursery to year 4) in mainstream schools. The majority of children referred to the team are year one pupils (4–5 year olds), attending schools in areas of low socio-economic status (LSES). There is evidence that for all of the referred children, teachers have identified concerns around their attention and language skills [32]. The RISE teams have developed a whole-class programme, known as the Attention and Listening Programme (ALP), that they currently provide to schools on request. This programme is similar to RECALL in its structure and format but it is not underpinned by WM theory and has not been robustly evaluated.

This protocol describes the rationale, methods and analysis plan for a proposed cluster randomised feasibility trial that will compare RECALL to the existing ALP intervention and education as usual. It aims to resolve uncertainties about the acceptability of RECALL to those who would be delivering it (health professionals and teachers), assess the feasibility of conducting a definitive cluster randomised trial (CRT) of its effectiveness and make a novel contribution to the WM literature regarding the barriers and facilitators to the implementation of WM training in real-life contexts.

### Study aim and objectives

The key research question is whether it is possible to design a definitive CRT that will evaluate whether RECALL is more effective than an existing intervention (ALP), and education as usual, for enhancing WM, attention and language skills in 4–5 year olds from LSES areas.

We will conduct a cluster randomised feasibility trial that will enable us to:

1. Examine the acceptability of the novel RECALL programme and its accompanying manual to the health professionals and teachers who deliver it.
2. Measure the implementation of RECALL by health professionals and teachers including compliance and fidelity of delivery.
3. Understand the trial processes at the cluster and individual levels including recruitment, consent and sampling procedures, attendance levels and loss to follow-up.
4. Determine the acceptability of randomisation to schools.
5. Explore the appropriateness of the existing intervention (ALP) as an active control comparator to the experimental RECALL programme.
6. Explore how WM, attention and language skills are typically supported in the classroom (education as usual).
7. Determine the appropriateness and acceptability of the outcome measures for the children, teachers and health professionals.
8. Identify the facilitators and barriers (at the cluster and individual levels) to the implementation of RECALL and refine the intervention’s logic model.^1^

## Methods/design

This study is a three-arm, cluster randomised feasibility trial with a parallel group design. The novel RECALL classroom programme will be compared to an existing intervention (ALP, the active control) and education as usual (the no intervention control). The delivery of the interventions at the classroom level necessitates the use of a cluster design for this study [34, 35] with each school constituting a cluster. The experimental RECALL programme and the active control intervention will be delivered by health professionals from the multi-disciplinary RISE teams once per week. Thus, they will demonstrate the programmes for the teachers who will then provide two further practice sessions during the week.

**Fig. 1:**
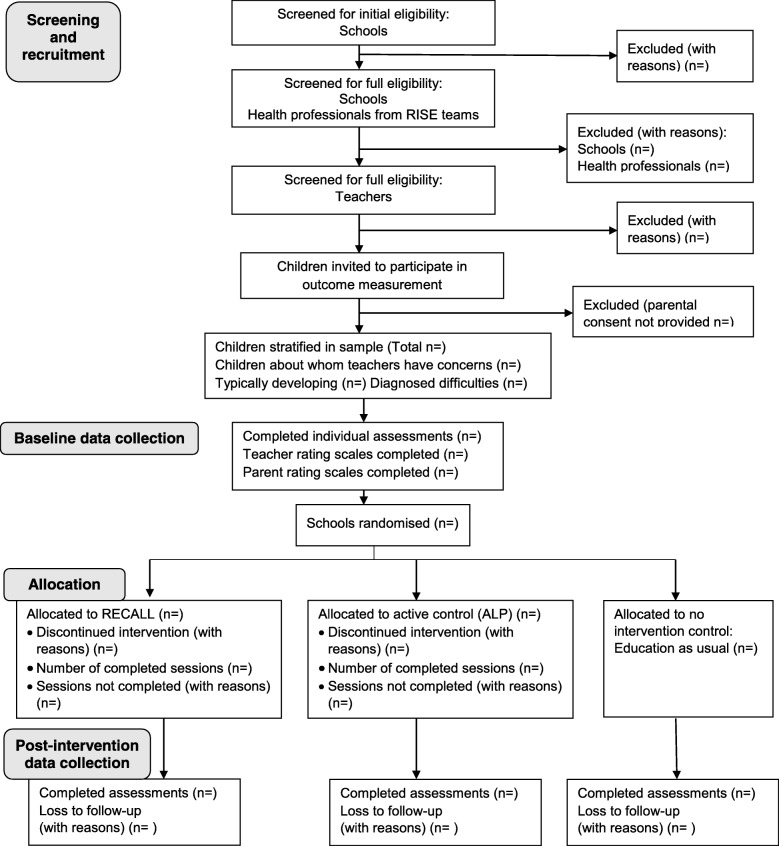
RECALL cluster randomised feasibility trial: protocol flow chart

**Fig. 2:**
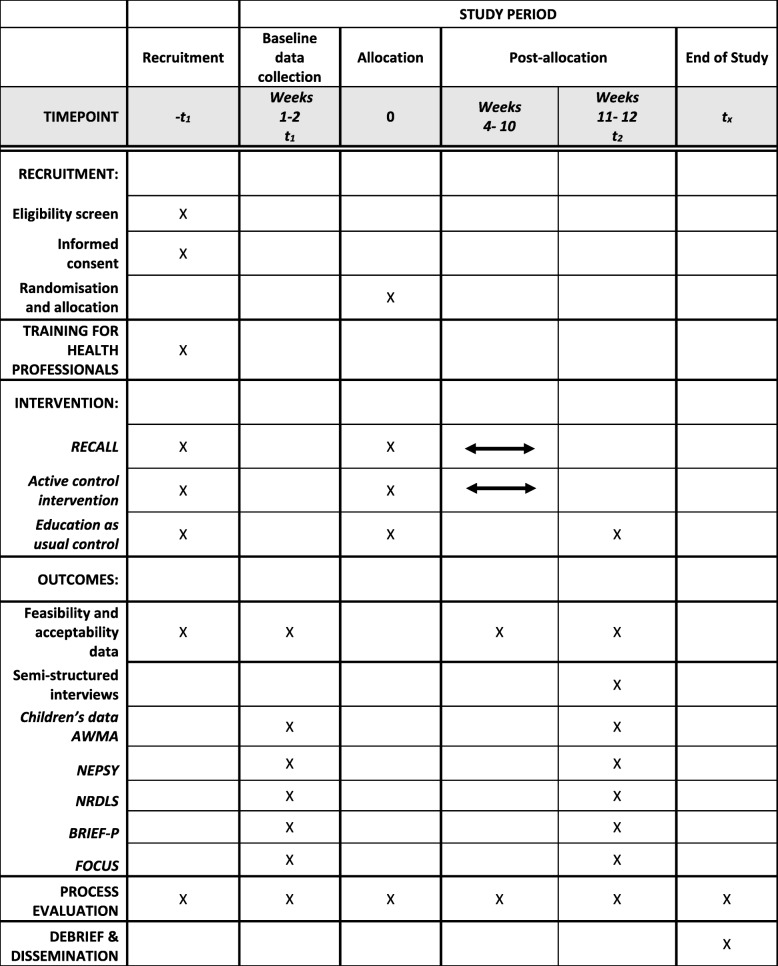
SPIRIT figure for RECALL cluster randomised feasibility trial

Children’s outcomes will be measured at two time points (baseline and 1-week post-intervention). The protocol has been developed according to the SPIRIT 2013 Statement [36] recommendations for protocol items for clinical trials and the CONSORT 2010 extension to cluster randomised pilot and feasibility trials [37]. Throughout the trial, a process evaluation will explore the factors that could impact on the internal and external validity of a future CRT and the intervention’s logic model for RECALL will be refined [38, 39]. Figure 1 shows the flow chart of the study and Fig. 2 illustrates the timing of all trial processes.

### Setting

This is a multi-site trial that will be conducted in primary schools in one region of the UK, Northern Ireland (NI), where children commence formal education at 4 years of age. The mainstream school population in NI includes a wide range of children including those with undiagnosed and diagnosed learning or developmental difficulties. Year one classes typically have one teacher, supported by a classroom assistant (CA). The multidisciplinary RISE teams are based within each of the five Health and Social Care Trusts (HSCT) in NI and this study will take place in two of the HSCT areas. The RISE teams work in partnership with schools and provide a range of services including: specialist assessment and intervention for referred children with recognised developmental difficulties; and targeted support for vulnerable children (considered to be at risk of developmental difficulties). Targeted interventions include whole-class and group programmes focusing on particular skills such as attention and listening. The aim of these interventions is to support all children in accessing the curriculum and reduce underachievement [40].

### Sample size and participants

As this is a feasibility study, and the purpose is to explore the acceptability of the intervention rather than its effectiveness, a formal a priori power calculation has not been conducted [41]. The results will not be used to estimate the sample size, intra-cluster correlation or treatment effects for a definitive trial because, in the case of cluster randomised feasibility trials, these can be unrealistic and misleading [42, 43]. Therefore, the number of clusters and individual participants to be recruited has been selected in order to assess the acceptability and feasibility of RECALL and the outcome measures for children’s WM, attention and language skills.

At the cluster level, six schools in areas of LSES will be recruited in total. One class of children in year one (*n = ~* 30) will participate in each school (total: *n =* ~ 180 children). Two classes will receive the RECALL programme, two will receive the active control intervention (ALP) and two will receive education as usual. In the schools allocated to the to RISE and ALP groups, all of the children in the participating classes will receive the interventions. Health professionals from the RISE teams (SLTs, OTs, PTs and SEBs) will be recruited to deliver the RECALL and active control interventions.

#### Stratification of children

At the individual level, ten children, their parents/guardians and teachers will be recruited in each class to complete the outcome measures (total sample: *n =* 60 children). Due to the age of the children involved in this study (4–5 year olds), the number of standardised measures available, particularly of WM and attention, is limited. To determine the appropriateness and acceptability of the outcome measures that have been selected, they will be trialled with children representing the typical range of ability in a year one class. Hence, we will use stratified purposeful sampling [44]. Teachers in each class will identify children in three sub-groups/strata: (1) children about whom they have concerns around listening and communication skills but do not have a diagnosed developmental or learning difficulty (*n =* 5); (2) children with diagnosed developmental or learning difficulties (*n =* 2); and (3) typically developing children who do not have any identified listening and communication problems as recognised by the teachers (*n =* 3 per class). Children considered to be typically developing, within areas of social disadvantage, will be included due to the high incidence of speech, language and communication needs in this population [3].

#### Recruitment strategy

To recruit clusters, areas of LSES will be identified using data from the Northern Ireland Multiple Deprivation Measure (NIMDM) 2017 [45]. The NIMDM ranks the super output areas in NI from the most to the least deprived across seven types (or domains) of deprivation. An initial scope of this data indicated that there are approximately 72 primary schools in LSES areas in the two HSCTs where this study will take place. These schools will be ranked according to the Education, Skills and Training domain of the NIMDM and the aim is to recruit schools within the lowest decile for each HSCT area. Two local collaborators will be consulted in order to identify schools that meet the eligibility criteria (Table 1). Schools will be contacted in writing by the researchers, followed up by a phone call and a face-to-face meeting with the principal and year one teachers to discuss the study further. If more than one teacher in a school is interested in participating in the study, one class will be randomly selected.

**Table 1:** Cluster and individual participant eligibility criteria and rationale

Participant group	Inclusion criteria	Exclusion criteria	Rationale
Cluster eligibility (schools)	Situated in areas of LSES in the two participating HSCT areas. Have requested support from the RISE team in relation to children’s attention and language skills.	Schools with no separate year one class, i.e. all year one children are taught within a composite class with older/younger children.	RECALL was not designed for composite classes.
Education staff (teachers and classroom assistants)	Work with year one classes in a school which meets the above criteria	Previously accessed the active control (ALP) intervention.	Teachers who have previously received the active control may use strategies or activities from it in their practice which may contaminate the study findings.
Health professionals	Situated in the two participating HSCT areas. Must be SLTs, OTs, PTs or SEBs with experience in delivering whole-class programmes.	Health professionals from the teams in the three HSCT trusts involved in the co-production of RECALL.	Teams that were involved in the co-production of RECALL may be biased and this could threaten the internal validity of the study [46].
Children	Currently in a year one class, aged 4–5 years, in a school that meets the above criteria. They may have diagnosed or undiagnosed learning or developmental difficulties.	Children whose first language is not English will be excluded from being selected for outcome measurement.	The outcome measures being trialled in this feasibility study are not standardised for children whose first language is not English.

To recruit children and their parents/guardians for outcome measurement (*n =* 10 in each school), the teachers will send a user-friendly research information leaflet, participant information sheet (PIS) and consent form home with each child. Parents will return the signed consent form to their child’s teacher. From the list of children for whom consent is obtained, the teacher will choose ten pupils for outcome measurement according to the stratified sampling method. To enhance enrolment and retention in the study, parents who complete the communication skills rating scale at both the pre-and post-intervention time-points will be entered into a prize draw for a £100 supermarket shopping voucher.

To recruit the health professionals to deliver the intervention, the team mangers will identify the professionals who meet the eligibility criteria (Table 1) and disseminate the PIS and consent form. Staff will email the researcher to indicate their interest in the study and a meeting will be arranged to discuss this further and obtain written consent.

#### Eligibility criteria

Table 1 provides the inclusion and exclusion criteria for each participant group and the rationale for each prerequisite.

### Consent

At the cluster level, written consent for trial entry will be obtained from the school principals [35]. Individual consent for participation will then be obtained from all other participants [34]. The teachers, parents/guardians and children will be asked to take part without being explicitly told about which intervention they will receive. This means consent is not fully informed but it reduces the risk of selection bias and enhances the internal validity of the study [47]. The acceptability of this and its impact on recruitment will be examined in the process evaluation. Consent from the health professionals and school staff will be obtained during the pre-study meetings with the first author. Regarding children’s participation in the study, parental consent will be obtained. Child assent is deemed to be inappropriate in this study due to the age of the children. Although some 5-year-old children may be able to provide assent [48], there are considerable challenges in knowing if this is accurate [49]. The amount of autonomy children typically exercise in a given situation is also an important consideration [50]. The RECALL programme will be delivered as part of the educational curriculum and in this context children typically have limited autonomy. Although children will not assent to take part, parents will be asked to inform their children that the study is taking place and will be given a child-friendly leaflet to help them tell their child about the research. If parents do not return the consent form, the child will still receive the RECALL or active control interventions but they will not be part of the research study and no outcome measurement will be carried out with them.

### Randomisation and allocation

The six schools will be randomised to each arm of the trial: two will receive RECALL; two will receive the active control intervention (ALP); and two will receive education as usual. This will be conducted by the schools’ names being placed in opaque, sealed envelopes which will be selected by an independent person from within the lead researchers’ institute. This process has been deemed as introducing a low risk of bias in allocation concealment [51]. Randomisation will occur after baseline data collection with the children. As previously indicated, children will be selected for outcome measurement using stratification. The teacher will sort the names of all those whose parents provide written informed consent into the three strata previously specified. If more than the required number in each sub-group consent, the participants will be randomly selected using the same process of placing names in opaque envelopes.

### Interventions

The experimental, theoretically underpinned RECALL programme and the active control interventions both incorporate group and whole-class activities designed to be fun for young children. The programmes are comparable in their structure, format and dosage (intervention frequency and duration). They both consist of six, 40-min sessions that are repeated three times per week for 6 weeks (18 sessions in total). The first session each week will be delivered by the health professionals who will model the activities for the teachers so that they can deliver the two further practice sessions.

#### Experimental intervention: RECALL

RECALL is a theoretically underpinned, multi-component, manualised intervention that explicitly targets WM skills in 4–5 year olds to enhance attention and language skills. It is underpinned by individual change theory (why the intervention components are expected to benefit WM and produce near- and far-transfer effects) and systems theory (considering the role of the school context in affecting change) [52–55]. It was co-produced through a series of workshops with one group of teachers, parents and health professionals in an inter-sectoral partnership [54].

##### Individual theory of change

The tasks included in RECALL are based on evidence from a recent systematic review [56]. The review found certain tasks designed to either target WM directly (listening recall and odd-one-out) or indirectly (cognitively-demanding physical activity, inhibition, phoneme awareness and fantastical play) produced improvements on WM and some benefits for near-transfer activities [57–67]. The common ingredient across the effective interventions was predominantly the executive-loaded nature of the trained task, i.e. training on a task that taps into attentional and processing resources under executive control and not just the storage of information [56]. It has been suggested that repeated practice on executive-loaded working memory (ELWM) tasks (rather than practising storage-only, short-term memory tasks) may improve the efficiency of processing or perhaps even facilitate the storage of information in WM [58, 68]. Based on this evidence, all of the components in RECALL are executive-loaded tasks, where attention must be divided between the storage and processing demands of the task. This may overlap with the way attention is used in everyday (real-world) activities. Hence, since treatment effects are more likely to transfer from trained activities to activities with overlapping features [26], the direct training on ELWM tasks in RECALL may benefit untrained WM tasks and real-world skills including attention and language.

In addition to directly training ELWM skills, RECALL includes two other tasks that were identified in the systematic review as having potential. Interventions targeting phoneme awareness skills [64, 65] and fantastical play [66] were found to impact WM indirectly. These are included in RECALL due to their associations with functional language outcomes and attention. They are complementary to and consistent with the theory that ELWM tasks support WM because they are also executive-loaded tasks, i.e. they tap into processing and attentional resources under executive control. Phoneme awareness is the ability to isolate and manipulate sounds in spoken words [69]. Improving phoneme awareness might enhance the phonological mechanisms underlying WM, through increasing the efficiency of processing and supporting the creation of accurate, structured phonological representations. This may in turn support language development since our ability to recall words depends on their phonological representations in long-term memory [70, 71].

Fantastical play is a type of pretend play that is fantasy-oriented [66]. For example, pretend play could involve pretending to make tea, whereas fantastical play might involve pretending to make swamp tea for a giant. Engaging in fantastical play may support children’s WM and other executive functions which are used to support switching between fantasy and reality and remembering the rules/scripts of the pretence [72, 73].

##### Systems theory of change (intervention delivery model)

The theory underpinning how the ELWM tasks in RECALL can be delivered in the classroom was developed through the co-production workshops. The socio-ecological model [74] was used as a framework to inform the development of an intervention logic model (i.e. a pictorial representation of the relationships between the required resources, activities needed, mechanisms of change and desired outcomes [33]). This supported the identification of multi-level factors that may impact on the programme delivery in the classroom, e.g. the timing of intervention sessions in the classroom, the availability of resources and staff training needs. Consequently, the theory underpinning the delivery of RECALL is that the co-designed tasks will work in the classroom context when staff are supported with adequate training and a detailed manual. The health professionals model one RECALL session per week in the classroom. The teachers follow the activity plan in the RECALL manual so that they become familiar with the tasks and can deliver them independently a further two times in the week. Detailed session plans and accompanying materials such as picture stimuli and worksheets are provided in the RECALL manual.

##### RECALL components

The ELWM tasks incorporated into RECALL include direct training on two ELWM tasks (listening recall and odd one out [58]), phoneme awareness training [64, 65] and fantastical play [66]. Table 2 provides a description of each task.

**Table 2:** RECALL components and task progression

RECALL component (ELWM task)	Task progression
Listening recall (direct WM training)	
- This task targets verbal ELWM. - The children listen to a short sentence, judge whether it is true or false and recall the last word of the sentence.	The number of to-be-remembered words increases from one word in week one to two words by week 6.
Odd one out (direct WM training)	
- This task targets visuospatial ELWM. - The children look at three pictures in a grid, decide where one the odd one out is (left, middle or right), then recall the location of the odd one out picture.	The number of to-be-remembered locations increases from one in week one, to three or four by week 6.
Phoneme awareness training	
There are four types of phoneme awareness task in RECALL, focusing on developing awareness of the initial sounds in words. 1. Alliterative matching: finding things that start with a target sound. E.g., “Book starts with ‘b’. Can you find the other things that start with ‘b’?” 2. Segmenting initial sounds: “what sound does ____ start with?” 3. Alliterative matching and blending the target to generate new words: “Find the one that starts with ___? Let us think of other things that start with _” 4. Blending sounds to identify words: “Look at these pictures. Can you find the b – all?”	The four tasks develop from the easiest (alliterative matching) to the most difficult (blending sounds) [75]. The difficulty level of the practice items in each task progresses from early to late developing phonemes based on typical speech sound development [76].
Fantastical play	
There is no direct training on fantastical play in RECALL. This is integrated into the programme through the use of a fantastical theme for each session, e.g. superheroes. However, the direct ELWM and phoneme awareness tasks incorporate the theme of each session throughout i.e., the words and pictures used relate to the theme.	

##### Task progression

The manipulation of WM loads on a trial-by-trial basis may be important for improving WM where research has particularly shown the value of adaptive training, i.e. task difficulty that increases or decreases automatically based on an individual child’s performance [77]. As RECALL is to be delivered in the group context, individual adaptive profiles cannot be rolled out. Instead, the programme is designed to become progressively more difficult across its 6 weeks. Table 2 shows how the direct ELWM and phoneme awareness tasks in RECALL progress in difficulty across the course of the 6-week programme. Due to two novel features of the RECALL trial (the age of the children and the group nature of the intervention), establishing the span level at which to commence training and how to increase the level of difficulty from week to week was reasoned from previous studies with older children [58]. The appropriateness of this for 4–5 year olds will be explored in this feasibility trial.

##### Dosage

The amount and intensity of training is often poorly reported in WM studies [56].^2^ The dosage to be implemented in RECALL is based on the best available evidence from the systematic review which indicated that 11 trials (practice items) of each direct ELWM task (listening recall and odd one out) delivered three times per week for 6 weeks was effective [58]. For the phoneme awareness tasks, the dose (number of trials) administered in previous interventions is unclear. However, several studies identified that training sessions lasted 10–15 min [64, 65] and this is replicated in RECALL.

##### Structure of RECALL sessions

**Fig. 3:**
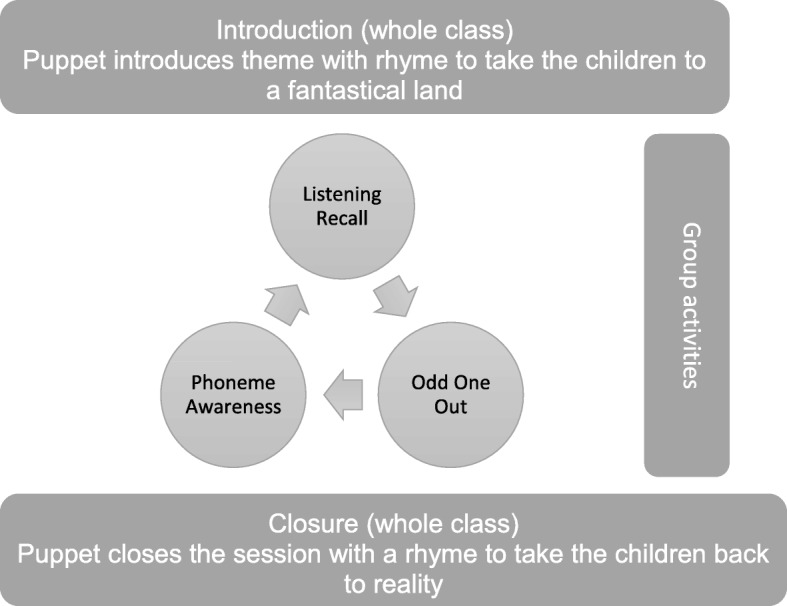
Structure of RECALL session

Each of the six RECALL sessions follows the same format, incorporating whole-class and group activities (Fig. 3). The fantastical theme for the week is introduced using a puppet who tells the children they are going on an adventure. The children are encouraged to enter into the fantasy by moving like characters in the fantastical land, e.g. posing like a superhero. The children are then divided into three groups that rotate around the direct ELWM and phoneme awareness training activities. The sessions close with another whole-class activity so the puppet takes them back to the reality of the classroom.

#### Active control group: existing ALP

The active control group in this study will receive an existing intervention (ALP) developed and used by the RISE teams. This has been selected as the active control condition because the programme is delivered in the same way as RECALL, i.e. the health professionals model the activities for the teachers and provide them with session plans so that they can replicate them. The programme also has the same dosage as RECALL (six, 40-min sessions that are repeated three times per week delivered once by the health professionals and twice the teachers). This recognises the importance of equating the training intensity between groups in WM research [24, 79].

The crucial difference between this programme and the experimental RECALL intervention is that the ALP intervention is not underpinned by WM theory. It aims to improve children’s attention and listening skills through repeated practice at listening tasks (e.g. the facilitator reads a story and the children must shake a musical instrument when they hear a particular word). It focuses on teaching children the importance of listening and on the use of visual, verbal and behavioural strategies to support listening, e.g. proximal praise. The tasks do not require the children to recall verbal or visuospatial information and they are not all executive-loaded. Whether this intervention is sufficiently different to act as a comparator in a full CRT will be explored in this trial.

#### No intervention control: education as usual

The two schools in this arm of the trial will not receive any whole-class interventions such as RECALL and the active control intervention during the 6-week trial period. Their teachers will be delivering the education curriculum as usual. How the teachers in these classes support the children’s attention and language skills during the study period will be explored in semi-structured interviews at the end of the trial.

### Compliance and fidelity

To enhance the implementation of RECALL, the health professionals, who will be modelling the programme each week for the teachers, will attend a 2-day training course prior to delivering it. They will be given the detailed programme manual which they will also supply to the teachers. To monitor compliance with the programme delivery and dosage, the health professionals and teachers will be asked to keep a simple log of their implementation (e.g. how often they delivered the programme and how long for). To monitor fidelity, three RECALL sessions in each school (one delivered by the health professional team and two by the teacher) will be observed by the first author. One of the health professional sessions and one of the teacher-delivered sessions will be observed simultaneously by a second member of the research team who will independently rate the delivery. Agreement between the raters will be checked by a third member of the team.

The researchers will use a structured observation tool designed within Carroll et al.’s (2007) [80] conceptual framework to explore four key elements of implementation (content, coverage, frequency, duration) and four moderating variables (intervention complexity, facilitation strategies, quality of delivery and participant responsiveness). Since this is a feasibility study, some adaptation to the intervention will be allowed (i.e. the researchers may address any problems raised by the health professionals or teachers). Any advice given will be recorded and examined in the process evaluation [81]. The interventions will be discontinued if a teacher withdraws from the study. If a health professional withdraws, another member of staff will be recruited and trained.

### Blinding

The school participants (principal, teachers and classroom assistants) and parents/guardians in the two intervention groups will be blinded to their group allocation. It is especially important that teachers and parents are blinded to group allocation, as their ratings of children’s attention skills will be used as outcome measures [25]. Maintaining the teachers’ blinding for the duration of the trial may be challenging, especially if the participating schools are in close geographical proximity to each other. This will be explored as part of the process evaluation in this study. Blinding of the health professionals to the schools’ allocation will not be possible as the teams will inevitably know which intervention they are delivering. The risk of this influencing the outcomes will be minimised further by the blinding of the research assistant (RA) who will be collecting the outcomes data. Whether the RA becomes aware of group allocation will also be investigated during the process evaluation.

### Outcome measures

Table 3 details the data that will be gathered at the cluster and individual levels to meet the primary objectives of this study regarding the acceptability of RECALL in the school setting and the feasibility of the trial processes.

**Table 3:** Acceptability and feasibility data at the cluster and individual levels

Data	Cluster level	Individual level
Acceptability of RECALL intervention and its manual to health professionals and teachers	Measures of compliance and fidelity.	Qualitative data: • Semi-structured interviews • Comments on intervention logs • Feedback from pre-study training provided for health professionals.
Compliance	Number of sessions delivered in each cluster	Qualitative data from semi-structured interviews including reasons for any sessions not being completed.
Fidelity	Structured observations by research team following Carroll et al. (2007) Research team records of any advice given.	–
Recruitment, consent and sampling procedures	Number and proportion of schools: • Meeting eligibility criteria • Approached • Principals who consent • Teachers who consent Number and proportion of children identified by teachers in each of the 3 sub-groups.	Number and proportion of parents who consent
Attendance levels and loss to follow-up.	Number of completed interventions	Number of standardised assessments, teacher rating scales and parent rating scales completed post-intervention
Acceptability of randomisation	School consent rates and reasons given for participation/non-participation.	Qualitative data: teachers’ perspectives on random allocation.
Acceptability of active control intervention as a comparator to RECALL	–	Qualitative data: health professionals’ perspectives on similarities/differences between the programmes. Observations of delivery by research team.
Exploration of education as usual	–	Qualitative data–semi-structured interviews with teachers in the education as usual control arm.
Acceptability of outcome measures for the children, teachers and RISE teams	–	Number of completed assessments for each child at each time point Number lost to follow-up and reasons why if possible Quality of audio-data will be reviewed Qualitative data: semi-structured interviews
Unexpected adverse effects	Any unanticipated effects will be recorded by the RISE team and teachers	
Blinding	Qualitative data: recording if blinding maintained at end of study.	

### Data collection

The acceptability and feasibility data will be gathered throughout the study and during the process evaluation (see Fig. 1). In WM research, it is essential to demonstrate clear causal pathways [25]. A future large-scale trial of the effectiveness of RECALL would therefore have to include a range of measures of children’s outcomes including measures of the trained activities (WM and phoneme awareness skills), untrained WM tasks (near-transfer) and attention and language skills (far-transfer effects). Testing the feasibility and acceptability of measuring all of these skills with 4–5 year olds, for whom the range of standardised assessments is limited, is one of the key objectives of this feasibility trial. This will occur at two points, baseline and post-intervention (see Fig. 2). Four types of measurement will be trialled with the sample of ten children in each cluster.

1. Standardised assessments administered directly with the children by a trained research assistant (RA).
  The RA, who will be blinded to group allocation, will withdraw children individually from their classroom for approximately one hour at each time point to complete the following standardised assessments:

- Phoneme awareness: The Preschool and Primary Inventory of Phonological Awareness (PIPA) [82]. This test is standardised for children aged 3 years to 6 years 11 months. It includes six subtests examining a range of phonological awareness skills. The phoneme isolation subtest will be used to directly assess children’s ability to identify the initial sound in a word, thereby providing a measure of a task directly trained in RECALL.
- Working memory: The Automated Working Memory Assessment (AWMA) [83] is a computerised assessment that will be administered using a laptop. This test has good validity as a measure of WM compared to other assessments [84]. Good test-retest reliability has also been demonstrated [15] and this measure is used widely in WM research. Two subtests will measure trained executive-loaded WM skills (listening recall and odd one out). Four further subtests will measure WM tasks not directly trained in the intervention (near-transfer effects). These subtests (digit recall, block recall, counting recall and non-word recall) have been selected due to their use in previous studies [58, 85].
- Attention: NEPSY-II–A Developmental Neuropsychological Assessment (NEPSY) [86]. Standardised, performance-based measures of attention for children under 6 years are limited [87]. The NEPSY-II is one of the few available assessments that includes attention subtests suitable for children of 4–5 years and (or its previous edition) has been administered in relevant studies [67].

2. Language: The New Reynell Developmental Language Scales (NRDLS) [88] is a standardised assessment for children aged between 3 years and 7 years 6 months. It has two scales: one that examines children’s understanding of selected vocabulary items and grammatical features (the Comprehension Scale); and another that tests children’s production of the same features of language (the Production Scale). This test is widely used in clinical and research contexts [89] in the identification of language impairment. It uses objects (rather than picture stimuli) during the assessment and this should make it accessible to the target population in this study.
3. *A teacher rating scale of the child’s attention in the classroom:* The Behaviour Rating Scale of Executive Function-Preschool Version (BRIEF-P) [90]. This tool is designed to specifically measure the behavioural characteristics associated with executive function skills including WM. It is a standardised, validated scale consisting of 63 items that can be used with children from 2 years to 5 years 11 months. It has good clinical utility and sensitivity and has been shown to complement performance-based measures of executive functions including WM [91, 92].
4. *A parent rating scale of the child*’*s language and communication skills at home.* The Focus on Communication Outcomes Under Six (FOCUS) [93]. This has shown excellent test-retest reliability and internal consistency [94]. The forms will be posted to the parents one week before each data collection point and they will be asked to return the form to their child’s teacher. The completed forms will be collected by the RA along with the teacher-completed BRIEF-P.
5. *Weekly monitoring of the child’s performance on the trained tasks.* Capturing individual responses will be an important part of a large-scale CRT due to the need for WM training to be adaptive. Therefore, assessing the feasibility of measuring children’s progress from week to week is an important part of the current feasibility study. For the classes receiving RECALL, the children’s performance on the direct WM and phoneme awareness tasks will take place during the first session each week which is delivered by the RISE NI teams. It is anticipated that the presence of additional adults in the classroom will facilitate this process. Each child will have a RECALL booklet in which they will complete the tasks using stampers or by drawing circles around the target pictures to indicate their responses. This will not be possible for some tasks that require purely verbal responses. For these tasks, digital voice recorders will be used (with parental consent) to record what the children say. There are significant uncertainties around the feasibility of audio-recording such data amidst the background classroom noise. Due to the level of uncertainty around this method, we aim to carry this out with just five, randomly selected children, in one of the RECALL classes. The voice recorder will be placed in a small gadget-holder which the children will wear across their bodies. A tie-clip microphone, connected to the recorder, will be placed on the children’s jumpers or lapels. The devices will be switched on and off by the teacher or classroom assistant.

### Data analysis

The feasibility data will be analysed and reported descriptively using means, frequencies and percentages. The data will be summarised and presented graphically. A CONSORT flow diagram will be used to report the response, recruitment and retention rate of clusters and individual participants at each point of the study. Where available, reasons for attrition and loss to follow-up will be reported.

Regarding the data obtained from the outcome measures completed with the children, statistical significance of treatment effects will not be analysed as this study would be under-powered for this purpose. However, between group comparisons will be conducted to inform the statistical model for the future trial. This will include two elements: (1) a series of one-way analyses (ANOVAs) of the baseline data of children’s standardised scores on the working memory (AWMA), attention (NEPSY subtests) and language measures (CELF-P) to investigate pre-intervention group differences; and (2) a series of analyses of covariance (ANCOVAs) to investigate post-intervention group differences). As the standardised assessments of WM, language and attention will be repeated within a short time-frame, results from the education as usual group will be used to examine test-retest effects. Qualitative analysis of the data gathered in the semi-structured interviews will be carried out using Braun and Clarke’s (2006) thematic analysis [95].

### Process evaluation

A process evaluation will be conducted throughout the study to support an understanding of how the trial processes relate to the context within which RECALL will be implemented [80]. The cluster and individual level data on the acceptability and feasibility of RECALL will be integrated with the qualitative findings and observations of cluster characteristics using the model proposed by Grant et al. [96]. Through this, the barriers and facilitators to implementation will be explored and the intervention logic model developed during the co-production phase will be refined.

### Criteria for proceeding to a full CRT

The primary factor for consideration as to whether to proceed to a full trial will be the feasibility data pertaining to recruitment and retention rates and the completion of outcome measures. However, strict thresholds for progression have not been set as these factors can be influenced by contextual variations that may not impact on a future trial [42, 97]. Rather, the decision to proceed to a main trial will be made along by the research team in collaboration with the Trial Steering Committee. Solutions to any problems observed in the feasibility trial will be sought through four potential options suggested by Bugge et al. [98]: (1) adapt the intervention, (2) adjust the context within which the intervention would be delivered, (3) amend elements of the trial design or (4) implement a combination of all of these actions.

### Monitoring

#### Trial steering committee

The conduct of this trial will be overseen by a Trial Steering Committee (TSC) that includes experienced researchers, key stakeholders from the health and education sectors in NI and service users. This group has been involved in the design of the trial and will meet at an interim point to review the progress towards the trial aims and to monitor the safety and well-being of all participants. Due to the nature and purpose of the primary data to be collected in this study (feasibility and acceptability measures), a Data Management Committee is not deemed necessary at this stage but will be established prior to the definitive CRT.

#### Modifications

Any modifications to the study protocol such as the eligibility criteria, recruitment procedures or outcome measures will only be carried out in agreement with the TSC. The changes made and reasons underlying them will be carefully recorded as these could be vital to the design of the future trial.

#### Stopping guidelines

Any adverse events occurring in the course of the trial will be carefully recorded and reported to the TSC, although this is not anticipated given the low risk nature of the study. The TSC will be contacted in this unlikely event and the trial may be discontinued. The study may also be discontinued if both schools in the RECALL arm of the trial withdraw. In the case of the trial being discontinued, all of the other active participants will be informed. The active control intervention may be continued as part of routine practice by the health professionals but no further data would be collected from the children for outcome measurement. The parents would be informed accordingly.

#### Ethical considerations

As noted, this is a low-risk study in that no additional potential harm is associated with the research compared to the everyday activity of the participants. The main ethical considerations relate to the welfare of children, parental consent and the children’s capacity to assent, and the maintenance of confidentiality. The schools’ policies regarding health and safety and child protection will be obtained in advance by the research team and adhered to throughout the trial. All members of the research team will hold valid Access NI Enhanced Disclosure Certificates^3^ confirming they are permitted to work with children in compliance with NI legislation. Through the whole-class sessions, it is possible that children with neurodevelopmental difficulties may be identified by the health professionals. In this instance they will advise the teacher of an appropriate service to which the child could be referred and ask them to seek parental consent prior to making a referral. Children who already attend another service will continue to receive this support during the trial. The procedures for obtaining parental consent in this study have been designed according to guidance provided by the Health Research Authority (2017) [50] (see consent section). All information/data obtained during the study will remain confidential and will be held in accordance with the General Data Protection Regulation (EU) 2016/679. Aside from the initial consent forms, all further material will be identified by unique number only, with no identifying information. The procedures used to ensure anonymity and confidentiality may be subject to audit through Ulster University’s annual audit programme.

#### Dissemination

The findings from this trial will be disseminated to all of the participants at a local level through informal networks in the first instance. At a national and international level, the results will be disseminated through conferences and publications in professional literature and peer-reviewed journals prior to the final design and conduct of a full CRT.

## Discussion

To our knowledge, RECALL is the first theoretically underpinned, multi-component, whole-class intervention that specifically aims to enhance WM, attention and language skills in 4–5-year-old children through activities applied within their everyday context. This study responds to calls for ecologically valid approaches to WM intervention for young children [28, 99] and the testing of creative approaches for children with low-language ability who are at risk of academic underachievement and poor employment prospects [8, 9].

A realist perspective underpins all stages of this research, influencing the intervention development, trial design and process evaluation. This approach recognises the impact of context on the implementation of interventions in real-life settings [39, 52, 53], which has been lacking in WM research to date. Therefore, the findings from this feasibility study will be of interest to researchers and practitioners interested in implementing WM interventions in the classroom.

In particular, the observations of RECALL being delivered in the classroom will allow investigation of whether it is possible for WM interventions to be delivered in the classroom with fidelity to their design and dosage. Key in this examination will be whether the executive-loaded nature of the trained tasks is maintained in their implementation. The inclusion of qualitative data in the process evaluation is a strength of this feasibility trial. This will allow us to explore participants’ views on the acceptability of RECALL and, crucially, the reasons for any differences between the intervention model and their fidelity to it. Thus we will be able to identify issues that may impact on the external and internal validity of a large-scale CRT.

The study design takes cognisance of the previous criticisms of the methodological quality of previous WM research [23–25], through the inclusion of an active control group (receiving a comparable intervention in terms of structure and dosage) and the blinding of outcomes assessors. The findings from this feasibility study will inform whether it can be scaled-up into a full CRT to evaluate the clinical and cost-effectiveness of RECALL.

## Trial status

Recruitment will commence in December 2018.

## Abbreviations

ALP: Attention and Listening programme
CA: Classroom assistant
CRT: Cluster randomised trial
HSCT: Health and Social Care Trust
LSES: Low socio-economic status
NI: Northern Ireland
OT: Occupational therapist
PT: Physiotherapist
RA: Research assistant
RECALL: Recall to Enhance Children’s Attention Language and Learning
RISE: Regional Integrated Support for Education
SEB: Social Emotional and Behaviour Specialist
SLT: Speech and language therapist
STM: Short-term memory
TSC: Trial Steering Committee
WM: Working memory

## Footnotes

1 See Kellogg Foundation 2004 guidance for a comprehensive overview of logic modelling [33].

2 The term dosage is used to refer to all components of the intervention intensity; dose refers to the number of trials per single intervention session [78].

3 AccessNI Criminal Record checks are compulsory for all employees or volunteers in working with vulnerable adults or children in NI (https://www.nidirect.gov.uk/articles/types-accessni-checks)
